# Polymicrobial Anaerobic Meningitis Detected by Next-Generation Sequencing: Case Report and Review of the Literature

**DOI:** 10.3389/fmed.2022.840910

**Published:** 2022-02-22

**Authors:** Xiaoqiang Li, Hui Du, Zhibin Song, Hui Wang, Xiong Long

**Affiliations:** ^1^Department of Neurology, Affiliated Xiaolan Hospital, Southern Medical University, Zhongshan, China; ^2^Department of Clinical Laboratory Center, Affiliated Xiaolan Hospital, Southern Medical University, Zhongshan, China; ^3^Emergency Department, Affiliated Xiaolan Hospital, Southern Medical University, Zhongshan, China

**Keywords:** metagenomic next-generation sequencing, anaerobic meningitis, polymicrobial infection, case report, nervous system infection

## Abstract

**Background:**

Anaerobic meningitis is a severe central nervous system infection associated with significant neurological sequelae and high mortality. However, the precise detection of causative pathogen(s) remains difficult because anaerobic bacteria are difficult to culture. Next-generation sequencing is a technology that was developed recently and has been applied in many fields. To the best of our knowledge, the use of next-generation sequencing for cerebrospinal fluid analysis in the diagnosis of anaerobic meningitis has been rarely reported.

**Case presentation:**

Here, we report a case of polymicrobial anaerobic meningitis diagnosed using next-generation sequencing of cerebrospinal fluid in a 16-year-old girl. Five species of anaerobic bacteria (*Porphyromonas gingivalis, Prevotella enoeca, Campylobacter rectus, Fusobacterium uncleatum*, and *Actinomyces israelii*) were detected by next-generation sequencing and treated with antibacterial agents (ceftriaxone, vancomycin, and metronidazole). The patient responded well to antibacterial treatment. Further inspection revealed bone destruction at the base of the skull, which further confirmed that these bacteria had originated from the oral cavity. One month later, the patient's condition improved significantly. At the same time, we performed a literature review on anaerobic meningitis using studies published in the last 20 years.

**Conclusions:**

This case emphasizes the importance of applying metagenomic next-generation sequencing to clinch the clinical diagnosis for patients with central nervous system infection. Metagenomic next-generation sequencing has been reported to be an important diagnostic modality for identifying uncommon pathogens.

## Background

Anaerobic meningitis is an uncommon disease. However, its true incidence may be underestimated because anaerobic bacteria in cerebrospinal fluid are difficult to isolate and culture; therefore, the prognosis of anaerobic meningitis is usually poor (1, 2). Despite treatment, the mortality rate of patients with anaerobic meningitis may be as high as 30.8% (3). Reliable laboratory tests performed early in the disease course are essential for the diagnosis and treatment of anaerobic meningitis.

Anaerobic bacterial culture of cerebrospinal fluid (CSF) is not performed routinely because cases of meningitis caused by anaerobic pathogens are rarely encountered (4). In addition, anaerobic bacterial culture is difficult to perform. Instead, polymerase chain reaction (PCR) to amplify the 16S ribosomal RNA (rRNA) gene is often used to detect the anaerobic pathogens causing bacterial meningitis. However, PCR can only detect designated pathogens via specific probes and targeted primers, due to which many pathogens may be missed (5, 6).

Unlike traditional testing for specific pathogens, metagenomic next-generation sequencing (mNGS), an emerging and promising modality, can identify a wide variety of potential causes (bacterial, viral, tuberculosis, fungal, and parasitic) (7). Improving our ability to identify novel or unexpected pathogens (8, 9). It also has the advantages of low cost and rapid turnaround time (10).

Although anaerobic meningitis is being increasingly recognized and reported in recent years, rapid identification of the causative anaerobic pathogen using mNGS resulting is improved patient outcomes is rarely reported. Here, we present a case of a patient with anaerobic meningitis. Multiple CSF cultures performed initially remained negative; however, the patient was finally diagnosed with polymicrobial anaerobic meningitis secondary to sinusitis when five species of anaerobic bacteria were isolated using mNGS. In addition, we reviewed the main features of the reported cases of anaerobic meningitis published in recent years.

## Case Presentation

A 16-year-old girl presented to the emergency department due to complaints of fever, severe headache. Her physical examination revealed a fever of 38.1°C; and neurological examination revealed a stiff neck and positive Brudzinski and Kernig signs. The Glasgow Coma Scale score was 10.

Head computed tomography (CT) showed brain swelling. A lumbar puncture was performed, which revealed a high opening pressure (310 mmH_2_O). CSF analysis showed that the fluid was cloudy, having a high protein (544.1 mg/dL, reference range 15–45 mg/dL) and low glucose (0.88 mmol/L, reference range 3.3–4.5 mmol/L) content and an elevated white blood cell count with neutrophilic predominance (13,206 cells/mm3, polymorphs 80%). Meanwhile, her CSF sample was sent to laboratory for pathogen detection at low temperature. Briefly, the patient's parents had signed informed consent, the CSF sample was collected and stored at −20°C, and then sent to laboratory of BGI-Shenzhen within 12 h. DNA was extracted with a TIANamp Micro DNA Kit (DP316, TIANGEN BIOTECH, Beijing, China) following the manufacturers' instructions. DNA libraries were constructed via end-repaired adaptation added overnight, and application of polymerase chain reaction amplification to extracted DNA. A Qubit dsDNA HS Assay Kit (Thermo Fisher Scientific Inc.) in combination with quantitative PCR was used to quantify DNA libraries. DNA sequencing was then performed on the BGISEQ-50 platform (BGI-Shenzhen, Shenzhen, China) (11). High-quality sequencing data were generated after filtering out low-quality, low-complexity, and shorter reads. Then, the remaining sequencing data were aligned to the Date of BGI, which contains 6,350 bacteria, 1,798 DNA viruses, 1,064 species of fungi and 234 parasites, to identify the pathogenic sequences. An advanced data analysis was then performed, as for the mapped data.

Our hospital laboratory test results showed leukocytosis with neutrophilia (total leukocyte count: 25.4 × 10^9^/L; neutrophils: 88.7%) and elevated levels of interleukin-6 (200.8 pg/mL) and procalcitonin (49.9 ng/ml). CSF and blood cultures were performed multiple times. However, brain magnetic resonance imaging performed the following day was reported to be normal. Intravenous ceftriaxone 80 mg/kg once daily and vancomycin 50 mg/kg twice a day were administered. Mannitol 20% and steroids was also administered to lower the intracranial pressure.

**Figure 1:**
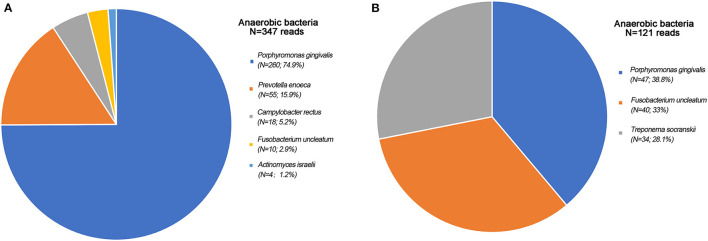
Anaerobic bacteria found by mNGS. **(A)** Anaerobic bacteria found by mNGS first times. Porphyromonas gingivalis: 260 reads; Prevotella enoeca: 55 reads; Campylobacter rectus: 18 reads; Fusobacterium uncleatum: 10 reads; Actinomyces israelii: 4 reads; **(B)** Anaerobic bacteria found by mNGS second times. Porphyromonas gingivalis: 47 reads; Fusobacterium uncleatum: 40 reads; Treponema socranskii: 34 reads.

Over the next 3 days, fever and headache resolved. However, the Kernig sign remained positive. Blood bacterial culture and gram staining of the CSF on admission was normal. We decided to perform further investigations (chest and abdominal CT and abdominal ultrasonography) to identify the source of infection. Unfortunately, these investigations did not yield positive results. Meanwhile, thorough oral cavity, dental, ear, nose, and throat examinations were performed to identify a potential source of infection; and no tooth decay or pathological changes were identified. mNGS results showed anaerobic bacteria, namely, *Porphyromonas gingivalis, Prevotella enoeca, Campylobacter rectus, Fusobacterium uncleatum*, and *Actinomyces israelii* (Figure 1), which are all oral bacteria. We thought it may be a mistake of the laboratory or contamination during CSF collection. And, repeat CSF cultures were negative.

**Figure 2:**
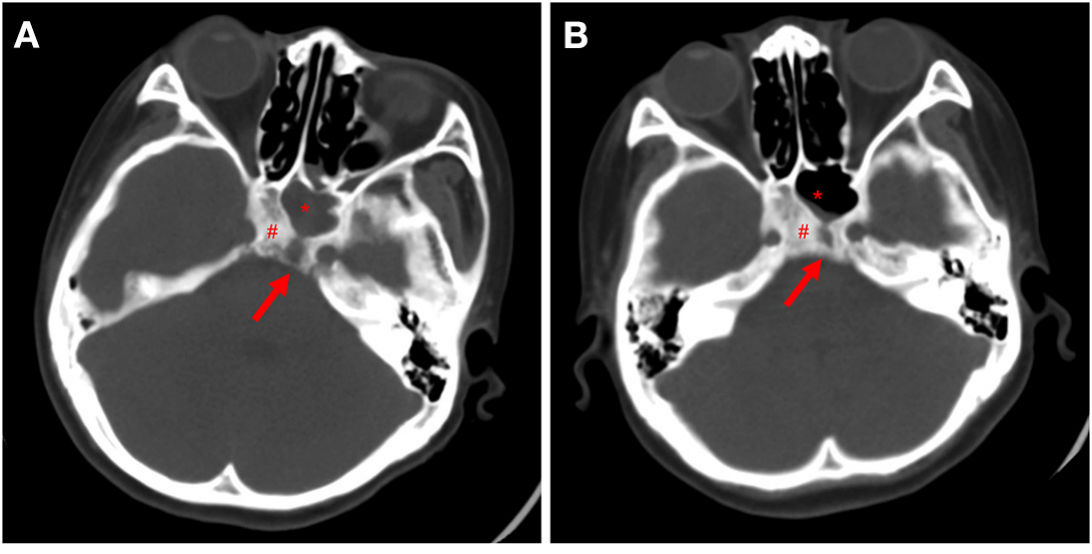
Paranasal sinus caused significant bony erosion. **(A)** Cranial CT at admission. **(B)** Cranial CT 1 month later. Red arrows indicate bone destruction. ^#^refer to the clivus, *for sphenoid sinus.

Lumbar puncture was performed again to detect the pathogens by mNGS 20 days later, which showed that the anaerobic bacteria were the same as before (Figure 1). At this time, we reviewed the patient's head CT findings (Figure 2) again and found that the patient's clivus, bony part of the skull base, was eroded. Repeat head CT (Figure 2) was immediately performed, and the bone quality was significantly better than that before admission. This patient had no history of cancer or trauma, and we speculated that sphenoid sinusitis had led to the destruction of clivus with subsequent infection of the meninges by oral anaerobic bacteria. Due to persistent elevation of white blood cell count on CSF analysis, we added metronidazole, which targets anaerobes, to the regimen. At 3-month follow-up, the patient appeared well and had returned to normal activity. Repeat head CT showed resolution of bone destruction (Supplementary Information).

## Discussion

Anaerobic meningitis is an uncommon disease occurring due to contiguous spread of infection from the head or neck (12, 13). In general, infections caused by anaerobic bacteria are usually devastating (14). Here, we report a case of polymicrobial anaerobic meningitis caused by oral anaerobic bacteria entering the intracranial cavity due to bone destruction of the base of the skull secondary to sinusitis. Moreover, mNGS identified the pathogen in time, and the patient received optimal antibiotics, which led to a good prognosis.

Currently, the diagnosis of bacterial infections relies on the isolation and culture of bacteria. Bacterial culture of CSF remains the gold standard for the diagnosis of bacterial meningitis. However, the diagnostic yield of CSF culture is low, and the process is time-consuming (requiring more than 72 h) (15–17). This may be the reason why the incidence of anaerobic meningitis is underestimated in clinical practice, since many of the pathogens causing intracranial infections are not identified (18, 19). In terms of treatment, when the causative pathogen cannot be identified, broad-spectrum empirical antibiotics are administered, which induces antibiotic resistance and increases the burden on the patients (20). Considering the difficulty in culturing anaerobes, PCR of CSF is employed to detect microbial DNA in patients with bacterial meningitis. The 16S rRNA gene is a DNA sequence encoding 16S rRNA. It is found in the bacterial chromosome, but does not exist in Non-prokaryotic organisms such as viruses and fungi. The 16S rRNA gene has a high degree of specificity and conservation. In the literature, most cases of anaerobic intracranial infections were definitively diagnosed using 16S rRNA PCR (14, 21, 22). However, this technique does not identify all bacteria and pathogens that are detected by CSF culture (21, 22). Moreover, the current PCR techniques used to detect bacterial meningitis are too expensive for patients in rural regions (23, 24). Thus, it is difficult to implement existing PCR tests in areas with the highest incidence of bacterial meningitis.

Therefore, rapid diagnosis of intracranial infections and preliminary classification of bacteria are clinical problems that need to be resolved urgently. mNGS is a novel and promising approach in diagnostic microbiology having the ability to detect many potential microorganisms using a single assay (7, 25). Previous studies show that mNGS of CSF obtained from patients with central nervous system infections improved the diagnostic rate and provided actionable information (26, 27). In this case, the mNGS detected the pathogens in time and provided a direction for us to identify the source of infection. Below, we review the relevant literature on anaerobic meningitis.

Recent studies on anaerobic meningitis that were published in the last 20 years were identified using an electronic search (Table 1). Our analysis showed that very few cases of anaerobic meningitis have been reported. However, the incidence of bacterial meningitis is very high worldwide (28, 29). The main reason for this may be that anaerobic meningitis is difficult to diagnose. The age at onset reported in these studies was variable, reflecting that anaerobic meningitis can affect a wide range of age groups. Moreover, the prognosis of the patients was very poor (21, 22), and only a few of the cases reported complete recovery of the patient (4, 30). However, the prognosis was poor compare to bacterial meningitis (31). Inappropriate antibiotic therapy administered due to the delay in diagnosis is the cause of increase in the sequelae and mortality of anaerobic meningitis (32).

**Table 1:** Main features of reported cases of anaerobic meningitis.

**Author**	**Bacteria**	**Methods of identification**	**Treatment**	**Type of pathogeny**	**Outcome**	**Sex/age (years)**
Kalay et al. (4)	*Bacteroides fragilis*; *B. thetaiotaomicron* and *Fusobacterium necrophorum*; *Proteus mirabilis*.	MALDI-TOF MS; Left ear for culture.	Vancomycin; Metronidazole; Meropenem; Acyclovir metronidazole	Mastoiditis	Recovery	M/16
Llitjos et al. (18)	*Peptostreptococcus micros, Fusobacterium necrophorum*, and *Porphyromonas gingivalis*	16S rRNA sequencing; standard culture.	Meropenem; Aancomycin; Fosfomycin; Amoxicillin; Metronidazole	NA	Death (47 days)	W/69
Anusha et al. (19)	*Bacteroides fragilis*	16S rRNA sequencing; standard culture.	Ceftriaxone; Amoxicillin; Acyclovir	A subdural empyema; Pre-sacral abscess.	Death	M/8-week
Yael et al. (12)	*Eubacterium multiforme*	16S rRNA sequencing;	Ceftriaxone; Vancomycin; Metronidazole; Ampicillin	Brain penetrating trauma	Neurological sequelae.	M/6
Joshua et al. (27)	*Anaerobic gram-negative bacillus*.	Blood culture	Benzylpenicillin;Metronidazole;	Rectothecal Fistula Arising from an Anterior Sacral Meningocele	Recovery	M/48
Juan at al. (28)	*Bacteroides fragilis, Staphylococcus aureus* and *Morganella morgagnii*	CSF culture	Vancomycin and meropenem	Colorectal surgery	Uneventful outcome	M/68

Exact incidence of anaerobic meningitis is unclear and was presented in only a few case reports. We found that PCR can be an important method for the diagnosis of anaerobic meningitis but it does not detect all organisms and is expensive. Moreover, culturing anaerobic organisms may be difficult; therefore, it is challenging to promptly diagnose anaerobic meningitis. This case shows that mNGS may be more effective than traditional microbial detection methods. In addition, early diagnosis and timely administration of appropriate antibiotic treatment can be life-saving. Efforts should be made to ensure the widespread availability and use of mNGS.

## Conclusions

We were able to correctly diagnose our patient with anaerobic meningitis, owing to the application of mNGS, due to which she was administered appropriate antimicrobial therapy. This case demonstrates that the process of diagnosing anaerobic meningitis is imprecise due to which its incidence may be higher than reported. Possibility of anaerobic meningitis should be kept in mind if the clinical course of the patient does not progress as expected and the mNGS technology may be a good tool to help establish the correct diagnosis.

## Data Availability Statement

The original contributions presented in the study are included in the article/Supplementary Material, further inquiries can be directed to the corresponding author.

## Ethics Statement

Written informed consent was obtained from the individual(s), and minor(s)' legal guardian/next of kin, for the publication of any potentially identifiable images or data included in this article.

## Author Contributions

XLi reviewed the literature, analyzed the patient data, and wrote the manuscript. HD and XLi were responsible for data collection. All the authors read and approved the final manuscript.

## Conflict of Interest

The authors declare that the research was conducted in the absence of any commercial or financial relationships that could be construed as a potential conflict of interest.

## Publisher's Note

All claims expressed in this article are solely those of the authors and do not necessarily represent those of their affiliated organizations, or those of the publisher, the editors and the reviewers. Any product that may be evaluated in this article, or claim that may be made by its manufacturer, is not guaranteed or endorsed by the publisher.

## Abbreviations

CSF: cerebrospinal fluid
PCR: polymerase chain reaction
CT: computed tomography
mNGS: metagenomic next-generation sequencing
rRNA: ribosomal ribonucleic acid.
