# Supplementary figures and images for: Polymicrobial Anaerobic Meningitis Detected by Next-Generation Sequencing: Case Report and Review of the Literature
Source: Front Med (Lausanne). 2022 Feb 22;9:840910. doi: 10.3389/fmed.2022.840910 (PMC8902384; doi:10.3389/fmed.2022.840910)

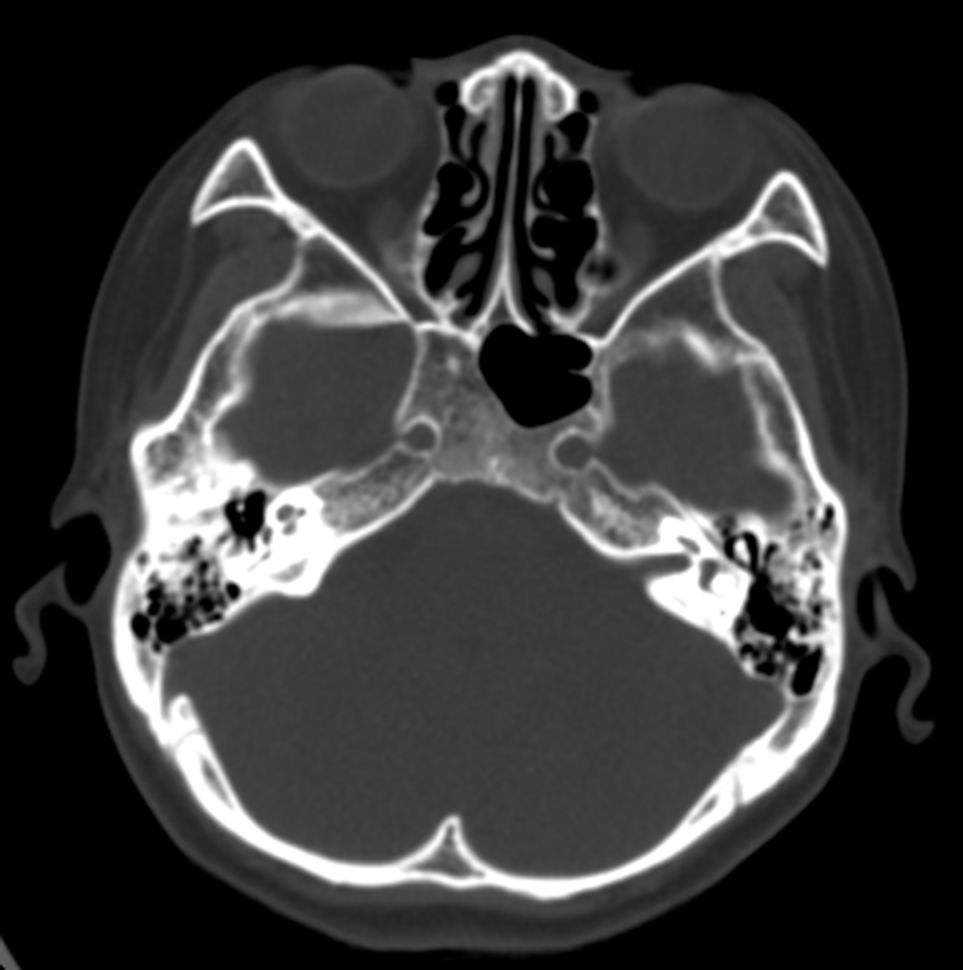

Supplement: Supplementary file 1 [file Image_1.TIF]
